# Peridynamic Simulation of Dynamic Fracture Process of Engineered Cementitious Composites (ECC) with Different Curing Ages

**DOI:** 10.3390/ma15103494

**Published:** 2022-05-12

**Authors:** Weiye Hou, Yuyang Hu, Chengfang Yuan, Hu Feng, Zhanqi Cheng

**Affiliations:** 1 School of Applied Science and Technology, Hainan University, Danzhou 570216, China; honbin713@126.com; 2 School of Civil Engineering, Zhengzhou University, Zhengzhou 450001, China; chengfang1102@126.com (C.Y.); fenghu@zzu.edu.cn (H.F.); zqcheng@zzu.edu.cn (Z.C.)

**Keywords:** crack propagation, early-age engineered cementitious composites, numerical simulation, peridynamics, time-varying laws

## Abstract

The mechanical properties of engineered cementitious composites (ECC) are time-dependent due to the cement hydration process. The mechanical behavior of ECC is not only related to the matrix material properties, but also to the fiber/matrix interface properties. In this study, the modeling of fiber and fiber/matrix interactions is accomplished by using a semi-discrete model in the framework of peridynamics (PD), and the time-varying laws of cement matrix and fiber/matrix interface bonding properties with curing age are also considered. The strain-softening behavior of the cement matrix is represented by introducing a correction factor to modify the pairwise force function in PD theory. The fracture damage of ECC plate from 3 to 28 days was numerically simulated by using the improved PD model to visualize the process of damage fracture under dynamic loading. The shorter the hydration time, the lower the corresponding elastic modulus, and the smaller the number of cracks generated. The dynamic fracture process of early-age ECC is analyzed to understand the crack development pattern, which provides reference for guiding structural design and engineering practice.

## 1. Introduction

To ensure adequate safety and reliability of buildings and to avoid disasters, in addition to structural design optimization, there are higher requirements for concrete materials. Professor Victor Li has developed engineered cementitious composites (ECC) made of disordered short fibers with significant strain-hardening properties [1]. As a new type of construction material, ECC can achieve an ultimate tensile strain of more than 3%, which overcomes the disadvantages of traditional concrete materials such as easy cracking and brittleness, and improves the durability and ductility of the structure [2]. Currently, many researchers have conducted more in-depth studies on ECC materials in the normal use phase. The fatigue resistance [3], penetration resistance [4], and corrosion resistance [5] of ECC are superior. ECC can also work cooperatively with concrete and steel bars [6]. Yun et al. [7] investigated the flow properties, fiber dispersion, and compressive, splitting tensile, bending, and uniaxial tensile properties of hybrid-fiber-reinforced cementitious composites. Cheng et al. [8] used recycled brick powder instead of quartz sand to study the mechanical properties of ECC. However, in the early age of ECC materials, its performance has significant differences compared with the normal use phase. The study of the force characteristics and the time-varying law of performance of ECC materials in their early age has important theoretical significance and practical value for the safety analysis and control during the construction period as well as the whole-life analysis.

A few researchers investigated the basic mechanical properties and strain hardening capacity of early-age ECC. Chan et al. [9] investigated the bond strength at the interface between PVA fibers and matrix at different ages by single fiber pull-out tests. Hu et al. [10] investigated the tensile mechanical properties of strain-hardened cementitious composites from 3 to 28 days. Azevedo et al. [11] explored the effect of açaí fibers on the hydration and microstructure of Portland cement pastes. Their research shows that the interfacial morphology of the fibers greatly affects the properties of the material. Using ECC as the adhesive between CFRP and concrete, Widanage et al. [12] studied the change of the mechanical properties of the specimens with time. Feng et al. [13] prepared ECC with excellent properties using local Chinese ingredients, and established a lattice discrete particle model (LDPM) considering the interaction between the fiber and the matrix. Previous studies illustrate the importance of studying fiber interface and curing age on ECC performance.

In addition to experimental studies, Victor Li et al. [14] improved the bonding and slip behavior of the interface between the fibers and the matrix by using micromechanical theory. Ellis B.D. et al. [15] developed a finite element model considering the interface transition zone and investigated the pullout response of different fiber morphologies. Zhang et al. [16] used the finite element method to predict the bonding behavior between different shapes of steel fibers and the matrix at different ages. Numerical simulation methods have emerged rapidly with the rapid development of computers, mainly including finite element method, extended finite element method and meshless method, etc. Numerical simulation can obtain results without much expense. The traditional finite element method requires additional fracture criterion to determine the location of crack emergence and expansion direction, and has certain mesh dependence. The extended finite element method gets rid of the mesh dependence in crack extension simulation [17], but still faces problems such as computational accuracy when studying three-dimensional problems or complex crack extension problems. The high order continuity function of the meshless method itself leads to its low computational efficiency.

Silling [18] proposed the theory of peridynamics (PD) in 2000, which is a non-local theory distinct from classical continuum mechanics. It solves the problem of crack initiation and propagation through the equation of motion in integral form, and has unique advantages in solving discontinuous problems. Since it fundamentally avoids the singularity caused by discontinuity problems and does not require the introduction of additional fracture criteria, it has been widely used for discontinuity problems such as damage failure of various materials. Gerstle et al. [19] were the first to apply peridynamics theory to the modeling of concrete structures, and the model explained the formation of macroscopic cracks at a microscopic level. Yaghoobi and Chorzepa [20] used a semi-discrete model to predict the crack path of fiber-reinforced concrete beams under bending loads. Gu et al. [21] implemented a contact impact algorithm to study the diffusion of elastic waves in split Hopkinson pressure bar (SHPB) tests and impact damage of the Brazilian disk. Hu and Madenci [22] presented a fatigue peridynamics model for composite laminates, and the progressive damage predictions were very close to the experimental results. Liu et al. [23] simulated the interaction of horizontal ice with a vertical cylindrical rigid structure at different velocities. Cheng et al. [24] simulated the dynamic brittle fracture of functional gradient materials (FGM) using bond-based peridynamics and investigated the effects of loading conditions and gradient modes on the properties of FGM. Chen et al. [25] proposed a peridynamic model based on the intermediate medium homogenization method to simulate the brittle damage of porous materials. Cheng et al. [26] investigated the effect of different angles of flaws on shale crack propagation using bond-based peridynamics. As the study of peridynamics theory has progressed in the past few years, more and more models have been applied to the theory of peridynamics. Yu et al. [27] established a micropolar bond-based model considering the tensile-rotational-shear coupling effect of bonds and used Timoshenko beam theory to establish the controlling equations of bonds to simulate the interaction between material points and the coupling effect of bonds. Zhang et al. [28] proposed an angle-based bond damage criterion for type II and mixed-mode fractures and developed a peridynamics model for mixed-mode fracture analysis, which was validated for fractures at mixed-mode interfaces and homogeneous materials. Han et al. [29] developed a dynamic mixed classical continuum mechanics and peridynamics medium model to study wave propagation in a linear elastic solid, and successfully simulated wave propagation and wave-induced crack nucleation. Wu et al. [30] investigated the fracture mode of laminated glass panels under drop weight loading by reconstructing a classical viscoelastic model within the framework of ordinary state-based peridynamics theory.

The previous studies have shown the great advantage of peridynamics in simulating crack propagation in cementitious composites. However, these studies are all qualitative or quantitative analyses of the properties of cement materials in the normal use phase and lack numerical simulations of the early age of these materials. The mechanical properties of cementitious composites exhibited at different ages vary greatly, which has a great impact on the construction schedule of the project. Considering the time-dependent behavior of bond stretch between material points in peridynamics, it can be used to model effects such as aging and fatigue [31]. In this study, a semi-discrete peridynamics model for ECC considering time-varying effects is proposed. The model is based on the study of Cheng et al. [32] and takes into account the degree of cement hydration and the variation of bond strength between fibers and matrix with age to characterize the effect of curing time on the strength of ECC. Uniaxial tensile and three-point bending simulations based on the PD model with time-varying effects were conducted on ECC plates at different curing ages to reveal the strength of ECC plates and the variation of crack initiation and propagation with age.

## 2. Review of Bond-Based Peridynamics Theory

**Figure 1:**
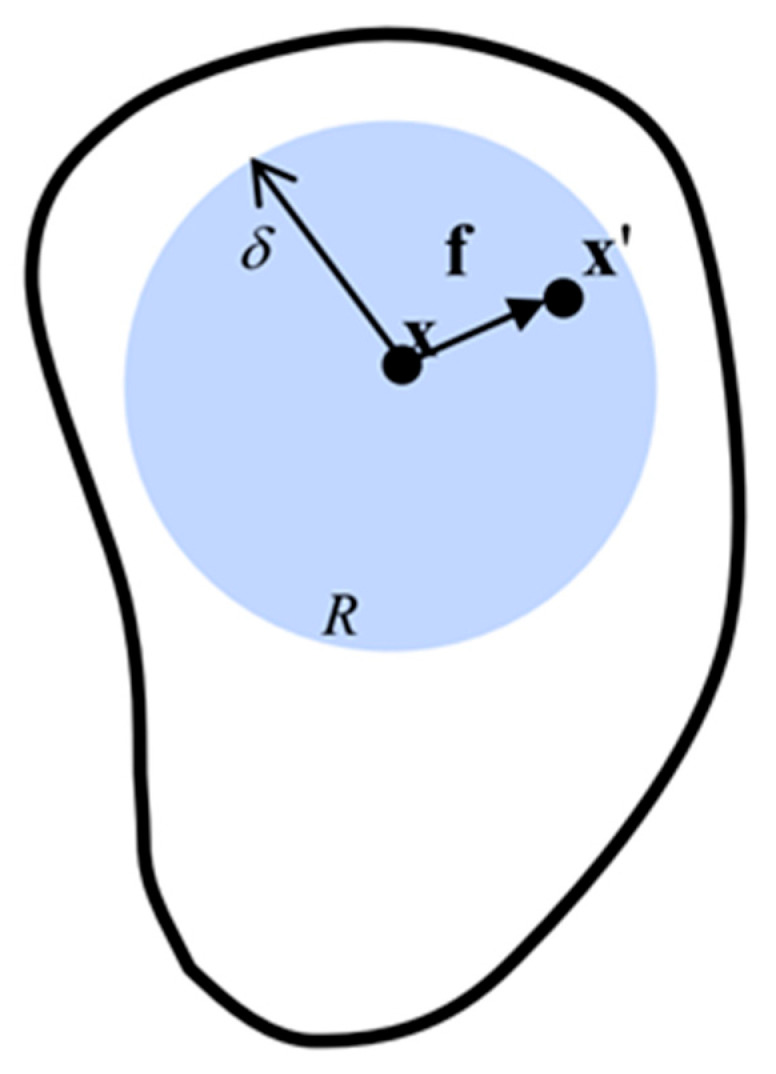
Interaction between point **x** and its family member **x**′.

Peridynamics [18,19] is a nonlocal continuum theory. It discretizes the material system into a multitude of material points containing all physical information, where each has a defined volume and density. There is an interaction force between the material point x and the material point $x^{\prime }$ within the radius δ of the horizon, as shown in Figure 1. At any time t, the equation of motion of the material point x can be expressed as

(1) $$\rho \ddot{u}\left(x,\;t\right)=\int_{H(x)}^{\;}f\left({u(x}^{\prime },\;t)-u\left(x,\;t\right){,x}^{\prime }-x\right){\mathrm{dV}}_{x^{\prime }}+b\left(x,\;t\right)\;\forall x\;\epsilon \;R,\;t\;\geq \;0$$

where ρ is mass density, u is the displacement of material point x, $H\left(x\right)$ is horizon region, the radius of the horizon region is δ. The interaction force between point **x** and point $x^{\prime }$ is denoted by f, which is called the pairwise force function. $b\left(x,\;t\right)$ is the body force density acting at time t in the material point x.

For simplicity, in the following discussion, the relative positions of the two points in the reference configuration are denoted by $\xi =x^{\prime }-x$. Their relative displacements are denoted by $\eta =u(x^{\prime },\;t)-u(x,\;t)$, and it is noted that ξ+η denotes the relative position vector between the points after deformation.

The interaction of material point **x** with other material points in its horizon is called bond, which is the fundamental difference between peridynamics and classical continuum mechanics. Here, the horizon can be understood in such a way that there is no interaction between these two points when **x**′ is not in the neighborhood of **x**, i.e.,

(2) $$\left|\xi \right|=\xi \;>\;\delta \;\Rightarrow \;f=0\;\;\forall \;\eta$$

According to [31], for micro-elastic materials, the pairwise force function **f** can be derived from a scalar micro-potential function w, i.e.,

(3) $$f\left(\eta ,\;\xi \right)=\frac{\partial w}{\partial \eta }\left(\eta ,\;\xi \right)\;\;\forall \xi ,\;\eta$$

where *w* denotes the energy stored in a deformed bond per unit volume squared, so that the energy contained in each unit volume of matter point is

(4) $$W=\frac{1}{2}\int_{H\left(x\right)}^{\;}w\left(\eta ,\xi \right){dV}_{\xi }\;\forall \eta ,\;\xi$$

Factor 1/2 appears because each endpoint of the bond possesses only half of the energy in the bond.

**Figure 2:**
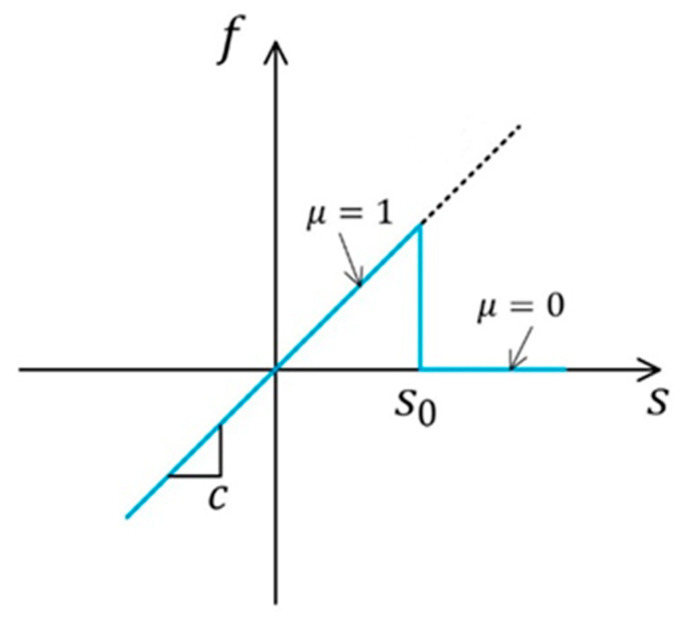
Relationship between force and bond stretch.

A linearized version of the pairwise force function for a micro-elastic material takes the following form, as shown in Figure 2. c(ξ) is a micromodulus function that needs to satisfy c(ξ)=c(−ξ), and the properties of *c* are discussed in detail in [18]. Where $s=\frac{\left|\xi +\eta \right|-\left|\xi \right|}{\left|\xi \right|}$ is the relative elongation of a bond, and for a homogeneous material under isotropic extension there is η=sξ.

(5) $$f\left(\eta ,\;\xi \right)=c\left(\xi \right)s\;\forall \eta ,\;\xi$$

Therefore, in the prototype microelastic brittle (PMB) material, the micropotential energy *w* is obtained according to Equation (3) as

(6) $$w=\frac{1}{2}{cs}^{2}\xi$$

Taking Equation (6) into Equation (4) leads to the strain energy density in peridynamics

(7) $$W=\frac{1}{2}\int_{H\left(x\right)}^{\;}{w\mathrm{dV}}_{\xi }=\frac{1}{2}\int_{0}^{\delta }\left(\frac{{cs}^{2}\xi }{2}\right){4\pi \xi }^{2}d\xi =\frac{{\pi cs}^{2}\delta^{4}}{4}$$

Letting Equation (7) be equal to the strain energy density obtained from classical linear elastic mechanics for the same deformation, the micromodulus *c* is obtained [33],

(8) $$\;c=\left\{\begin{array}{c} \;\frac{18k}{{\pi \delta }^{4}}\;\;\;\;\;\;\;\;\;\;3D\;\\ \;\frac{9k}{{\pi \delta }^{3}}\;\mathrm{plane}\;\mathrm{stress}\\ \;\frac{72k}{{5\pi \delta }^{3}}\;\mathrm{plane}\;\mathrm{strain} \end{array}\right.$$

Damage in peridynamics is characterized by the failure of bonds among material points, which happens when the elongation of bonds exceeds a critical stretch. When all bonds in a plane fail, a crack is formed. In the ideal case, the expression for the critical stretch $s_{0}$ can be obtained by equating the work required to completely separate the fracture surfaces with the critical energy release rate [33].

(9) $$s_{0}=\left\{\begin{array}{c} \sqrt{\frac{5G_{0}}{9k\delta }}\;\;\;\;\;\;\;\;\;\;\;\;\;3D\;\;\;\;\;\;\;\;\\ \;\sqrt{\frac{4\pi G_{0}}{9k\delta }}\;\;\;\mathrm{plane}\;\mathrm{stress}\\ \;\sqrt{\frac{5\pi G_{0}}{18k\delta }}\;\;\;\mathrm{plane}\;\mathrm{strain} \end{array}\right.$$

In order to describe the breakage of bonds between material points, it is necessary to introduce a judgment function $\mu \left(\xi ,t\right)$ with respect to time *t*.

(10) $$\mu \left(\xi ,t\right)=\left\{\begin{array}{c} 1\;\;\;\;\;\;\;\;\;\;\;\;\;\;\;s\left(t\right)<s_{0},t>0\\ 0\;\;\;\;\;\;\;\;\;\;\;\;\;\;\;other\;\;\;\;\;\;\;\; \end{array}\right.$$

At this point, the pairwise force function can be expressed as

(11) $$f\left(\eta ,\xi ,t\right)=\frac{\partial w(\eta ,\xi )}{\partial \eta }\mu \left(\xi ,t\right)\;\forall \eta ,\xi ,t\;>\;0$$

As can be seen from the above equation, the definitions of damage and fracture are included in the pairwise force functions of the peridynamics, and no additional failure criterion and stress intensity factor are required. Instead, they can be solved in a unified framework, which is where the peridynamics approach differs from the traditional numerical approach. The degree of damage φ at a certain material point can be expressed as

(12) $$\varphi \left(x,t\right)=1-\frac{\int_{H\left(x\right)}^{\;}\mu \left(x,t,\xi \right)dV_{\xi }}{\int_{H\left(x\right)}^{\;}dV_{\xi }}$$

When φ=0, it means that the material is in its original state; and when φ=1, it means that the bonds between the material point and all other material points in its neighborhood have been broken, i.e., the material point has failed.

## 3. Modified PD Model for ECC

### 3.1. Problem Presentation

Some scholars have already utilized peridynamics to model ECC and good results have been obtained. Reference [32] used a semi-discrete peridynamics model to successfully simulate the multiple cracking of ECC and the insensitivity to existing cracks. The fully discrete modeling approach proposed by Zhang and Qiao [34] adequately demonstrates the advantages and capabilities of peridynamics and fully discrete models in the simulation of tensile fracture behavior of fiber-reinforced cementitious composites. Previous studies have effectively demonstrated the feasibility of peridynamic theory in modeling the mechanical properties of ECC. However, the curing age also has an important effect on the performance of ECC, which has not been seen to be investigated by scholars using peridynamics theory.

### 3.2. Modified Cement Matrix Model

The constitutive model of PMB material is shown in Figure 2. Cheng et al. [32] modified the relationship between force and stretching of PMB materials by introducing a correction factor for bond damage to modify the pairwise force function. Equation (13) is function of the damage factor λ and the bond stretch s, the details of which can be found in [32,35].

(13) $$\lambda \left(s\right)=\left\{\begin{array}{c} \;1-\frac{s_{0t}}{s}e^{-\frac{s-s_{0c}}{s_{f}-s_{0c}}}\;\;\;\;\;\;\;\;s_{uc}\leq s<s_{0c}\\ \;\;\;\;\;0\;\;\;\;\;\;\;\;\;\;\;\;\;\;\;\;\;\;\;\;\;\;\;\;\;\;\;\;\;\;s_{0c}\leq s<s_{0t}\\ 1-\frac{s_{0t}}{s}e^{-\frac{s-s_{0t}}{s_{f}-s_{0t}}}\;\;\;\;\;\;\;\;s_{0t}\leq s<s_{0}\\ \;\;\;1\;\;\;\;\;\;\;\;\;\;\;\;\;\;\;\;\;\;\;\;\;\;\;\;\;\;\;\;\;\;\;\;\;\;\;\;\;\;\mathrm{others} \end{array}\right.$$

**Figure 3:**
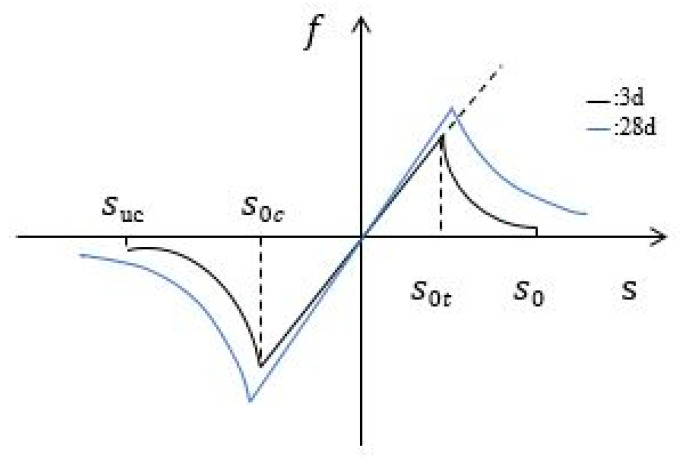
Constitutive function model of cement matrix material.

The performance of ECC at early age is influenced by the type and properties of the admixtures and the curing conditions. The elastic modulus of the early age cementitious material gradually increases with age and eventually tends to a constant value after the end of the hydration process. Instead of directly defining the amount of strain, peridynamics uses the elongation and shortening of bonds between material points to describe changes in the relative positions of the material points. In this study, a time-dependent function is introduced to describe the different properties of the bonds between material points at different ages. The peridynamics model of the cement matrix is shown in Figure 3. The pairwise force function in Equation (5) is improved to the form in Equation (14).

(14) $$f=\left(1-\lambda \right)\alpha c\left(\xi \right)s$$

(15) $$\alpha =\alpha_{u}\left(1-e^{\left(-\kappa t^{\chi }\right)}\right)$$

(16) $$\alpha_{u}=\frac{1.031\times \frac{W}{C}}{0.194+\frac{W}{C}}$$

where α is the variation law of the degree of hydration of the cementitious material with time [36]. κ is the time parameter, t is the curing age, χ is the shape parameter, $\alpha_{u}$ is the final degree of hydration, and $\frac{W}{C}$ is the water–cement ratio.

### 3.3. Modified Fiber–Matrix Interaction Model

**Figure 4:**
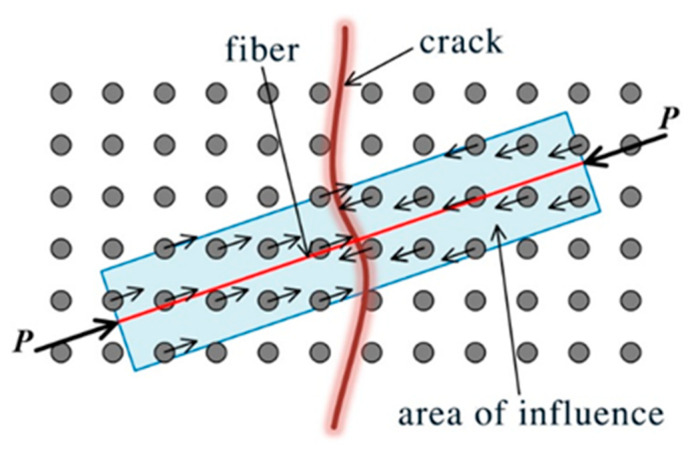
Semi-discrete fiber model.

ECC is an ultra-high toughness cementitious composite material with high tensile ductility. The degrees of freedom of fiber nodes often determine the computational efficiency and computational cost of the simulation process, so this study uses the semi-discrete fiber model proposed by Kang et al. [37] to solve the problem. The semi-discrete method is also effective in reflecting the various mechanical behaviors of fiber cementitious composites. This approach of distributing the fiber forces on the cementitious composite close to the fibers reduces the difficulties posed by the fiber node degrees of freedom during the simulation, as shown in Figure 4.

Lin et al. [38] proposed a model representing the force-pull-out displacement relationship for the fiber/matrix interaction as in Equation (17). The meaning of each letter can be found in [32,38].

(17) $$P\left(\Delta \right)=\left\{\begin{array}{c} \sqrt{0.5\pi^{2}E_{f}d_{f}^{3}\left(\tau_{0}\Delta \left(1+\eta \right)+G_{d}\right)}\;\;\;\;\;0<\Delta <\Delta_{0}\\ \pi d_{f}\tau_{0}\left[1+\beta \frac{\Delta -\Delta_{0}}{d_{f}}\right]\left(l_{e}-\Delta +\Delta_{0}\right)\;\;\;\;\Delta_{0}<\Delta <l_{e} \end{array}\right.$$

The mechanical properties of the cement matrix vary with time as the cement hydration process proceeds. However, the time dependence of the strength of the fiber/matrix bond interface differs from the time dependence of the mechanical properties of the matrix material due to the difference in microstructure. Chan et al. [9] investigated the variation of fiber/matrix interfacial bond strength and transition zone microstructure with hydration time. By single fiber pullout tests, they obtained the variation curves of fiber/matrix interface strength with age and the variation curves of pullout load versus pullout distance. On this basis, this study obtained the interfacial strength as a function of curing age by fitting the data

(18) $$Q=Q_{\infty }\left(1-e^{-\gamma t}\right)$$

Here, $Q_{\infty }$ is the fiber/matrix interfacial strength after completion of hydration, γ is the parameters related to fiber type, *t* is the curing age. Taking Equation (18) into Equation (17) we can obtain the relationship between force and tensile displacement at different ages, which can be written as

(19) $$P^{\prime }\left(\Delta \right)=\left\{\begin{array}{c} \sqrt{0.5\pi^{2}E_{f}d_{f}^{3}\left(Q\tau_{0}\Delta \left(1+\eta \right)+G_{d}\right)}\;\;\;\;\;0<\Delta <\Delta_{0}\\ \pi d_{f}Q\tau_{0}\left[1+\beta \frac{\Delta -\Delta_{0}}{d_{f}}\right]\left(l_{e}-\Delta +\Delta_{0}\right)\;\;\;\;\Delta_{0}<\Delta <l_{e} \end{array}\right.$$

### 3.4. Numerical Method

**Figure 5:**
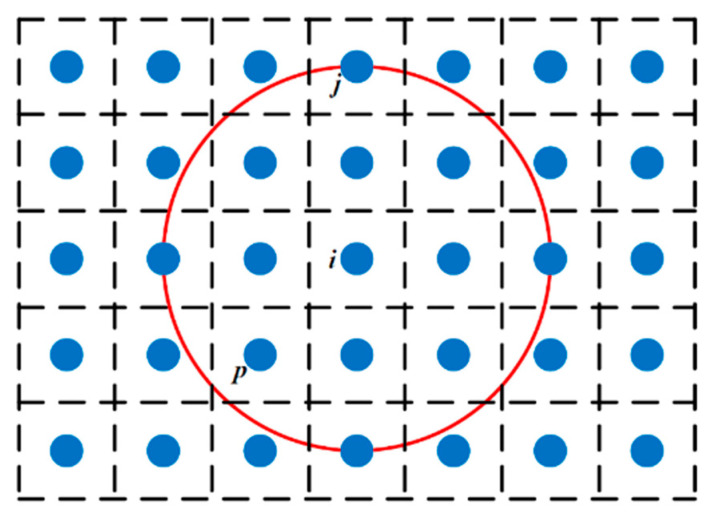
The discrete case of PD node and the horizon size of node i.

Peridynamics can consider discontinuous solutions. The whole model is discretized into material points defined in PD and a certain volume is assigned to each point. When the space and volume assigned to each point are uniform, the discretization is shown in Figure 5.

The basic equation of motion Equation (1) for PD can be rewritten using the finite summation method as

(20) $$\rho {\ddot{u}}_{i}^{n}=\sum_{p}f\left(u_{p}^{n}-u_{i}^{n},x_{p}-x_{i}\right)V_{p}+b_{i}^{n}$$

Here, n is the number of time steps, and the node number is represented by the subscript. $V_{p}$ represents the volume occupied by any point p in the horizon of point i. When integrating and summing the horizon of point i, the boundary node of the horizon is partially beyond the radius of the horizon, as shown in Figure 5. The volume of point j is only partially covered by the horizon of node i. In this case, in the two-dimensional plane state, the integral formula is modified by introducing a correction factor fac.

(21) $$\rho {\ddot{u}}_{i}^{n}=\underset{p}{\sum }f\left(u_{p}^{n}-u_{i}^{n},x_{p}-x_{i}\right)\left(fac\cdot V_{p}\right)+b_{i}^{n}$$

(22) $$fac=\left\{\begin{array}{c} \;\;\;\;\;\;\;1\;\;\;\;\;\;\;\;\;\;\;\;\;\;\;\;\;\;\;\;\;\;\;\;\;\;\;\;\;\;\;\;\;\;\;\;\;\;\;\;\;\left|\xi \right|<\delta -0.5\times +eps\\ \left(\delta -\left(\left|\xi \right|-\left(h/2\right)\right)\right)/h\;\;\;\;\;\;\;\left|\xi \right|<\delta +0.5\times h+eps\\ \;\;\;0\;\;\;\;\;\;\;\;\;\;\;\;\;\;\;\;\;\;\;\;\;\;\;\;\;\;\;\;\;\;\;\;\;\;\;\;\;\;\;\;\;\;\;\;\;\;\;\;\;\;\;\;\;\;otherwise\;\;\;\;\;\; \end{array}\right.$$

where h is the maximum node spacing. 

**Figure 6:**
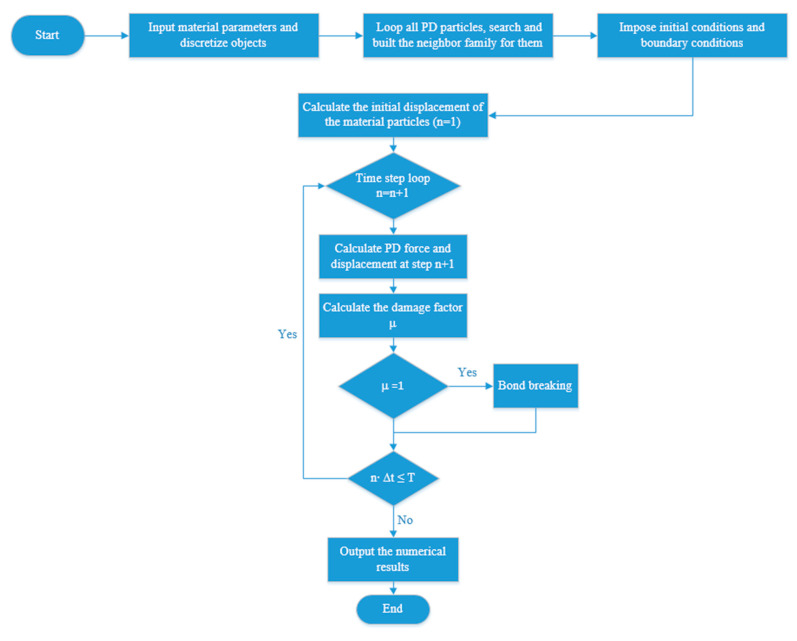
Flowchart of the PD program.

In this study, a central difference algorithm, namely velocity-Verlet algorithm, is implemented to solve the PD equations of motion

(23) $$\begin{array}{l} {\dot{u}}_{n+1/2}={\dot{u}}_{n}+{\ddot{u}}_{n}\Delta t/2\\ u_{n+1}=u_{n}+{\dot{u}}_{n+1/2}\Delta t\\ {\dot{u}}_{n+1}={\dot{u}}_{n+1/2}+{\ddot{u}}_{n+1/2}\Delta t/2 \end{array}$$

where, u, $\dot{u}$, and $\ddot{u}$ represent the displacement, velocity, and acceleration vectors, respectively. Δt is the time step. To obtain a stable solution of Equation (21), Δt should satisfy [31]:

(24) $$\Delta t<\sqrt{\frac{2\rho }{\sum_{j}V_{j}c}}$$

where $V_{j}$ and c represent the volume of point j and micromodulus between points i and j, respectively. The flowchart of the PD program used in this study is shown in Figure 6.

## 4. Numerical Examples

### 4.1. Simulation of ECC Plate under Uniaxial Tensile Stress

**Figure 7:**
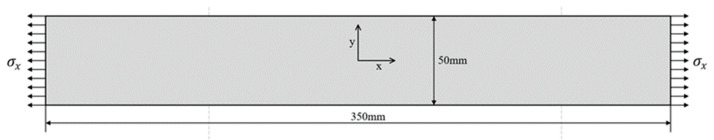
Specimen geometry and boundary conditions.

**Figure 8:**
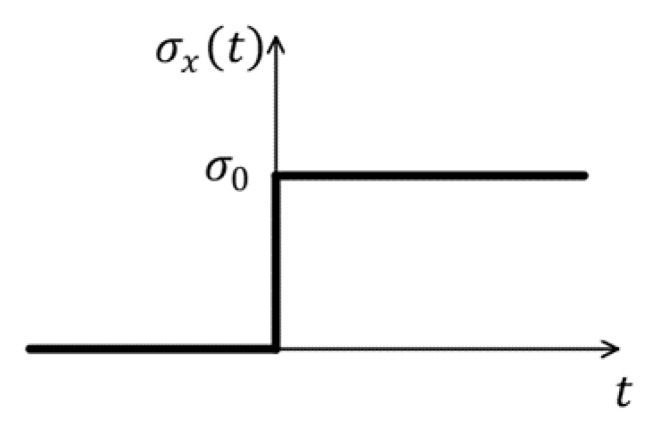
Variation of load with time.

**Figure 9:**
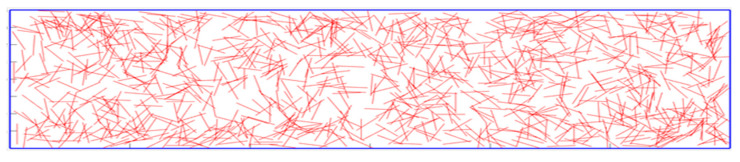
Fiber random distribution map.

The damage–fracture process of concrete materials can be typically reflected in the uniaxial tensile test. We consider a thin rectangular ECC plate with dimension of 350 × 50 mm under dynamic symmetric tension loads, as depicted in Figure 7. A sharp dynamic tensile load, $\sigma_{x}=\sigma_{0}=2\;\mathrm{MPa}$, which is observed in Figure 8, is symmetrically applied on the boundary of the ECC plate that is depicted in Figure 7. The material properties of the matrix and fibers in the model adopt the material parameters, as shown in Table 1 and Table 2, where $K_{IC}$ denotes fracture toughness. The fiber volume fraction is 2%. The random distribution of fibers in the matrix is shown in Figure 9. A uniform time step size of $\Delta_{t}=2\times 10^{-8}\;s$ is used. Considering the work done by Cheng et al. [32] on convergence, we choose the radius of horizon δ=2 mm, the distance between the material points $\Delta_{x}=0.5\;\mathrm{mm}$. In order to prevent sudden destruction of the boundary nodes when the traction condition is initially applied, we made some assumptions when writing the code. The bonds between the boundary nodes are considered indestructible, which means that they have a large critical stretch.

**Figure 10:**
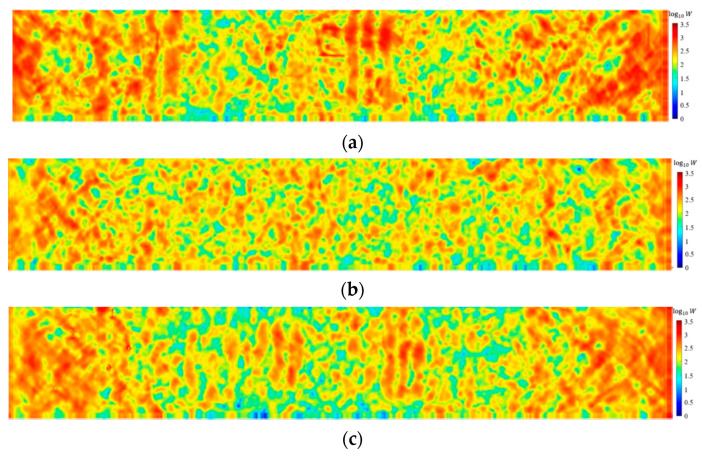
Stress clouds of specimens at different ages at 24 µs: (**a**) = 3 days; (**b**) = 7 days; (**c**) = 28 days.

Figure 10 shows the stress clouds of ECC plates at different ages when the load is applied for 24 µs. Reference [10] writes that the formulation of strain-hardened cementitious composites is based on two basic guidelines. First, the crack tip toughness $J_{tip}$ of the cement matrix must be less than the complementary energy $J_{b}^{\prime }$. Second, the first crack opening strength $\sigma_{fc}$ of the cement matrix is less than the maximum fiber bridging stress $\sigma_{0}$.

(25) $$J_{b}^{\prime }=\sigma_{0}\delta_{0}-\int_{0}^{\delta_{0}}\sigma d\delta$$

where $\sigma_{0}$ is the maximum fiber bridging stress and $\delta_{0}$ is the maximum crack opening.

The experimental data in Reference [10] shows that the complementary energy at 7 days is larger than that at 3 days and 28 days, which can be calculated according to Equation (25), i.e., the stress is lower at 7 days, and the simulation results in Figure 10 are consistent with this. The ductility of the cement matrix is greater at 3 days and the stress transfer is faster. However, fewer cracks appear because of the larger critical stretch. Although the stress at 7 days is less than that at 3 days, the critical stretch at 7 days is smaller, so it exhibits more cracks. The stress is higher at 28 dsys and the critical stretch is minimum, thus causing the most cracks. According to Equation (9), when the elastic modulus is small, the critical elongation $s_{0}$ of the material is large. This is the reason for the inconsistent number of cracks. As the cement hydrates, it gradually changes from ductile to brittle.

**Figure 11:**
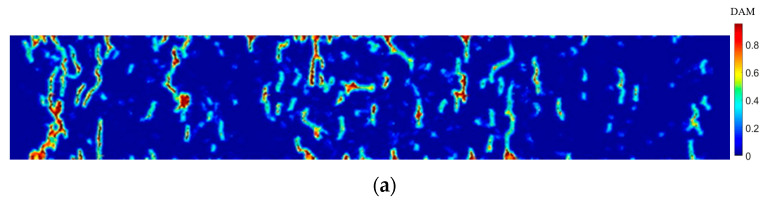

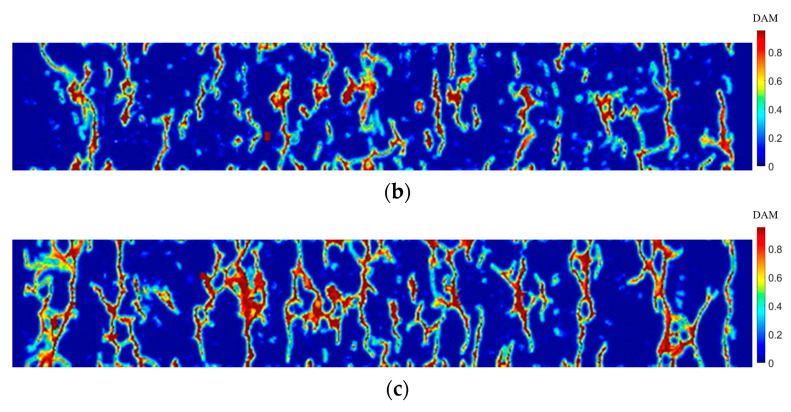
Uniaxial tensile crack propagation paths at different ages at 90 µs: (**a**) = 3 days; (**b**) = 7 days; (**c**) = 28 days.

The model proposed in this study was used for numerical simulation, and the crack propagation paths of ECC plates with ages of 3 days, 7 days, and 28 days at 90 µs were captured, as shown in Figure 11. The multiple cracking characteristics of engineered cementitious composites can be demonstrated. It can be seen from the graph that the number of cracks at 3 days are less than those at 7 and 28 days. On the one hand, it is because the hydration of cement is not yet completed at the early stage, and the cement matrix has a certain degree of ductility. Combined with the peridynamics theory, it is because the critical stretch of the bonds between the material points becomes larger and therefore exhibits less cracking when the force is the same. On the other hand, as the curing age grows, the fiber/matrix interaction changes from being mainly contributed by the interfacial frictional resistance to the bonding between the fiber and the interface. The hydration of the fiber/matrix interface transition zone deepens, the porosity decreases, and the tensile strength of the matrix material increases, reflecting the nature of gradually increasing brittleness with curing age. In terms of the number of cracks, those at 7 days and 28 days are already very close to each other, indicating that most of the hydration process has been completed at 7 days, which is in agreement with the actual situation [10].

### 4.2. Simulation of ECC Plate under Impact Load

**Figure 12:**
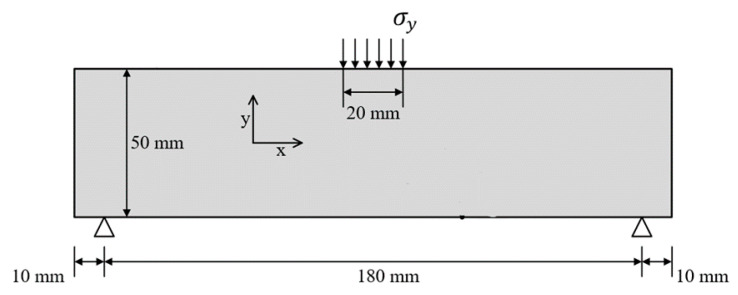
Specimen geometry and boundary conditions.

**Figure 13:**
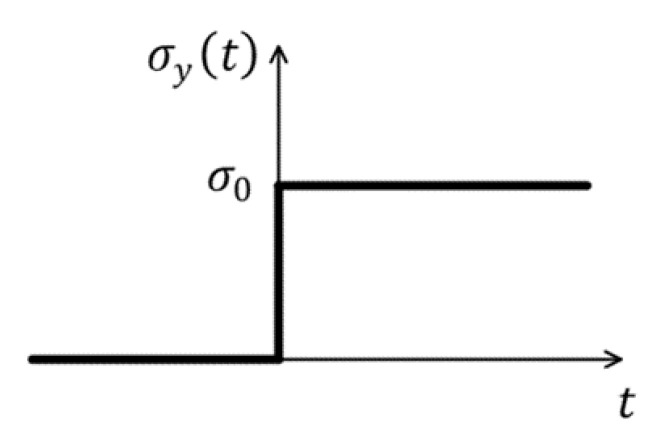
Variation of load with time.

In actual engineering structures, ECC as a protective reinforcement material exhibits good impact resistance when under impact loading. Previous studies on early-age ECC have focused on material properties, and there are fewer studies on mechanical properties such as damage and fracture. We consider a thin, rectangular ECC plate with dimension of 200 × 50 mm under dynamic impact load, as depicted in Figure 12. A sharp dynamic tensile load, $\sigma_{y}=\sigma_{0}=10\;\mathrm{Mpa}$, which is observed in Figure 13, is applied on the boundary of the ECC plate that is depicted in Figure 12. Material parameters and other conditions are the same as in Section 4.1.

**Figure 14:**
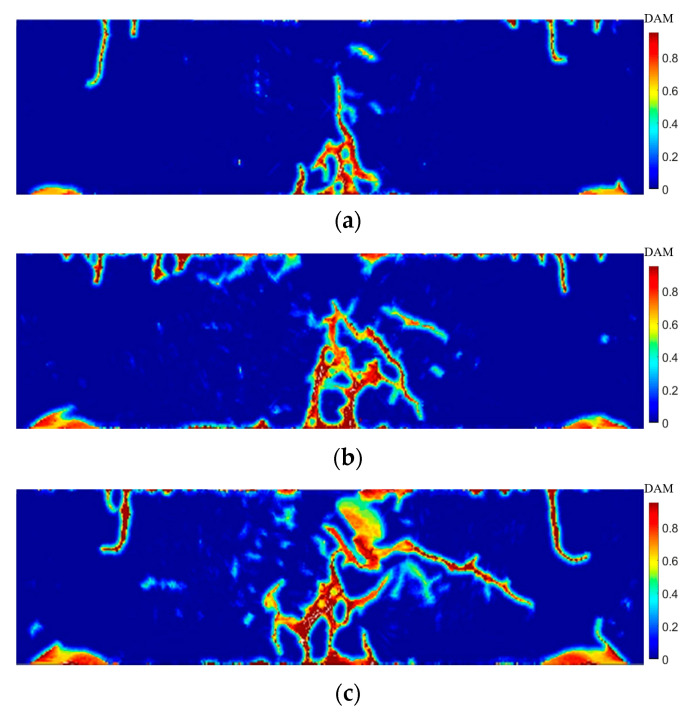
Crack propagation under bending load at 90 µs: (**a**) = 3 days; (**b**) = 7 days; (**c**) = 28 days.

Figure 14 shows the crack propagation paths for ages of 3 days, 7 days, and 28 days at 90 µs, respectively. Analysis of the cracking results shows that, due to the impact force, shear force was generated at the bearing and damage occurred at the bearing as well as at the upper part of the bearing. Cracking occurred at the center of the lower edge of the specimen, which was due to the stress reaching the condition of matrix cracking. The damage cracking was different at 3 days, 7 days, and 28 days. The example also shows that the ductility and critical stretch of the specimens differ for different curing ages. The brittleness of the specimens increases with the increase in the curing age. However, from the simulation results, it can be seen that the specimens of 3 days, 7 days, and 28 days have the characteristics of multiple cracking. From the perspective of PD, when the age is 3 days, the critical elongation $s_{0}$ of the bond is greater than 7 and 28 days, which means that the number of cracks in the age of 3 days is small. This study assumes that the fibers do not function until the bonds of the material points are broken, and that the interaction of the fiber/matrix interface is weak at a shorter curing age, so the crack width after the failure is large. This numerical example is a good verification of the ductility of the matrix material at early ages and also shows that the matrix parts can better share the external energy due to the fiber incorporation, showing multiple cracking.

## 5. Conclusions

By introducing the time-varying effects of early-age ECC materials into the peridynamics model and writing the corresponding codes, the crack propagation paths of ECC plates under uniaxial tensile and three-point bending loads at different ages were simulated. From the results of numerical simulations, the crack paths at different ages are basically in agreement with the experimental as well as the theoretical analysis results of the relevant literature. It also provides a powerful numerical tool for the analysis of damage fracture processes in early age cementitious materials. The proposed improved peridynamics model also provides a reference and some implications for future numerical modeling. From the engineering practice, in order to reduce the risk of cracking of ECC at an early age, firstly, the particle ratio should be appropriately selected to reduce the porosity. Secondly, as the fiber bridging effect cannot be ignored, fibers with stronger bonding to the cement matrix or fibers whose surface has been treated should be selected. It is worth noting that this study only presents the improved model and simulation results for the curing age before 28 days. Our next work is to study the results of longer curing age in order to better generalize PD theory and guide the application of ECC in engineering.

## Figures and Tables

**Table 1:** Mechanical parameters of the cement matrix.

𝐸 (GPa)	KIC (MPa·m12)	$\rho \;\left(Kg/m^{3}\right)$	$f_{t}\;\left(MPa\right)$
22.35	0.287	2380	5.36

**Table 2:** Mechanical parameters of fiber.

Fiber	$f_{tf}$ $\left(MPa\right)$	$d_{f}$ $\left(\mu m\right)$	$l_{f}$ $\left(mm\right)$	$E_{f}$ $\left(GPa\right)$	Elongation	$\tau_{0}$ $\left(MPa\right)$	$G_{d}$ $\left(J/m^{2}\right)$	β
PVA	1620	39	12	42.8	6%	2.0	2.7	0.5
